# Dual−Network PVA/PAM Hydrogel Strain Sensor for Machine−Learning−Assisted Rehabilitation−Oriented Hand Motion Monitoring

**DOI:** 10.3390/gels12080730

**Published:** 2026-08-17

**Authors:** Wendi Liu, Jintao Wang, Yuanduo Wang, Zhangqi Xia, Ruixin Liu, Yixuan Li, Xinyang He, Hailou Wang

**Affiliations:** 1 National & Local Joint Engineering Research Center of Technical Fiber Composites for Safety and Health, School of Textile & Clothing, Nantong University, Nantong 226019, China; 2 Shanghai Frontiers Science Center of Advanced Textiles, College of Textiles, Donghua University, Shanghai 201620, China; 3 Key Laboratory of Textile Science & Technology, Ministry of Education, College of Textiles, Donghua University, Shanghai 201620, China

**Keywords:** PVA/PAM hydrogel, double network, strain sensor, rehabilitation monitoring, machine learning, motion recognition

## Abstract

Wearable rehabilitation monitoring requires soft strain sensors with mechanical robustness, stable electromechanical responses, and intelligent motion recognition capability. Here, we report a poly(vinyl alcohol)/polyacrylamide (PVA/PAM) double−network hydrogel strain sensor for rehabilitation−oriented wearable monitoring. The hydrogel was prepared by ultraviolet ray (UV)−initiated acrylamide polymerization followed by freeze−thaw−induced PVA crystallization, forming a covalent PAM network interpenetrated with a physically crosslinked PVA network. The resulting hydrogel possessed a compact porous structure, improved stretchability, and stable deformation recovery. The optimized sensor exhibited a tensile strength of approximately 0.52 MPa, an elongation at break of approximately 480%, a response time of 0.12 s, and a recovery time of 0.17 s. It generated repeatable resistance signals under cyclic strain, finger bending, wrist motion, and grip training. Furthermore, the sensor enabled morse−code information transmission and support vector machine (SVM)−based recognition of rehabilitation−related hand states, including straight, bend, and clench. This work provides a soft hydrogel sensing platform for real−time rehabilitation−oriented hand motion, while morse−code encoding provides auxiliary assistance and an emergency communication function.

## 1. Introduction

Rehabilitation training requires continuous and quantitative assessment of joint flexion, hand grasping, wrist rotation, and repetitive limb exercises [1,2]. Accurate motion monitoring is important for evaluating training quality, patient compliance, and recovery progress [3]. Conventional rehabilitation assessment relies on therapist observation, camera−based systems, inertial units, or rigid electronic devices [4,5,6]. These approaches can suffer from limited skin conformability, motion artifacts, operational complexity, poor wearing comfort, and limited suitability for daily or home−based rehabilitation [7]. Soft wearable strain sensors that conformally attach to the body and convert biomechanical deformation into readable electrical signals are therefore desirable for personalized rehabilitation systems [8,9,10]. Hydrogel−based strain sensors are promising because of their tissue−like softness, high water content, stretchability, and tunable polymer networks [11,12]. Their mechanical compliance supports intimate contact with dynamic biological surfaces, while deformation−dependent resistance or impedance changes enable monitoring of joint motion and subtle physiological activities [13,14]. Conductive and ionically conductive hydrogels have consequently been explored in flexible sensors, electronic skin, human−machine interfaces, and soft robotics [15,16]. At the materials level, molecular regulation of conductive polymers such as PEDOT:PSS has shown that charge transport can be tuned through coordinated control of doping state and interchain interactions [17]. For rehabilitation−oriented sensing, conformal deformation with tissues and joints can reduce mechanical mismatch and improve signal fidelity during repeated motion [18,19,20].

Despite this progress, practical hydrogel strain sensors remain constrained by several challenges [21,22]. Single−network hydrogels often lack mechanical strength and fatigue resistance, which can cause damage or signal drift during repeated deformation [23,24]. Their high hydrophilicity can also induce swelling−related network relaxation and alter conductive pathways [25,26,27]. Conductive fillers or conductive polymers can improve conductivity, but they may cause aggregation, phase separation, or weak interfacial bonding [28,29,30]. These effects create a trade−off between electrical and mechanical performance. Such limitations are important in rehabilitation monitoring, which requires reliable operation during repeated and prolonged body movement [31,32,33]. Double−network hydrogels offer an effective solution by coupling an energy−dissipating network with a second network that preserves structural integrity during deformation [34,35,36]. In this context, polyacrylamide (PAM) can provide a flexible covalent framework, whereas polyvinyl alcohol (PVA) can form physically crosslinked microcrystalline domains and hydrogen−bonding interactions through freeze−thaw treatment [37,38]. Their combination is expected to produce a dense and robust hydrogel with improved stretchability, toughness, and recoverability [39,40], making it suitable for cyclic deformation during rehabilitation exercises [41,42,43].

Beyond material optimization, intelligent signal interpretation is essential for practical rehabilitation applications [44,45]. Hydrogel−derived motion signals are time−dependent, nonlinear, and influenced by movement amplitude, speed, and individual differences [46,47], whereas machine learning can extract discriminative features and convert these complex electrical responses into recognizable motion states [48]. Machine−learning−assisted hydrogel sensors have enabled object recognition, gesture classification, and human−machine interaction through analysis of strain or pressure induced electrical patterns. Integrating hydrogel strain sensors with machine−learning algorithms may therefore support data−driven recognition and analysis of rehabilitation−oriented hand motions [49,50].

Herein, we report a PVA/PAM double−network hydrogel strain sensor for rehabilitation−oriented wearable monitoring and machine−learning−assisted motion recognition. The hydrogel was constructed through ultraviolet ray (UV)−induced PAM covalent crosslinking and freeze−thaw−induced PVA physical crosslinking, producing a robust dual−network structure with stable electromechanical responses. The sensor showed gauge factors of 1.57, a response time of 0.12 s, and a recovery time of 0.17 s. It enabled real−time detection of finger, wrist, and hand rehabilitation movements; morse−code information transmission; and support vector machine (SVM)−based motion classification. This work provides an integrated material−sensor−algorithm platform for intelligent rehabilitation monitoring and machine−learning−assisted motion recognition.

## 2. Results and Discussion

**Figure 1:**
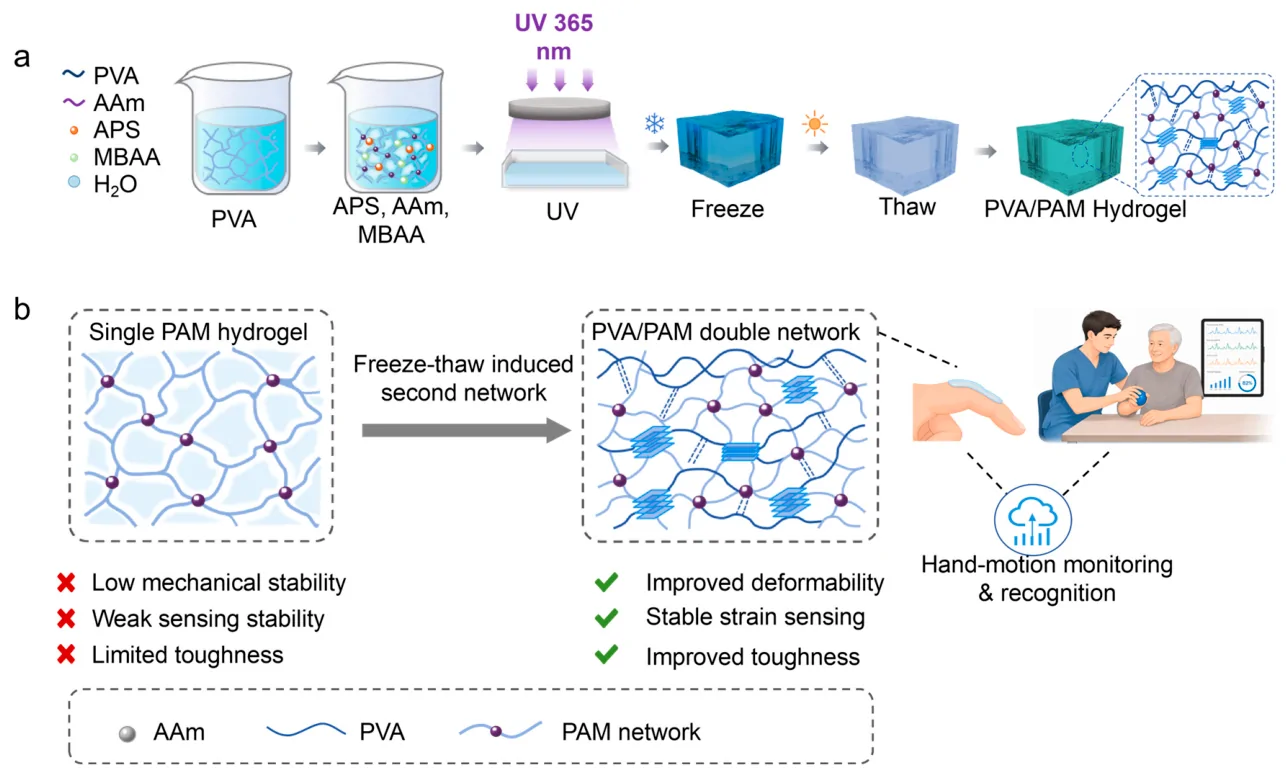
Design, fabrication, and rehabilitation concept of the PVA/PAM hydrogel sensor. (**a**) Fabrication process of PVA/PAM hydrogel. (**b**) Formation of the double−network structure and wearable rehabilitation monitoring.

To construct a soft sensing interface capable of following repetitive rehabilitation movements while retaining mechanical integrity and signal stability, a PVA/PAM double−network hydrogel is prepared through sequential covalent and physical network formation. As shown in Figure 1a, PVA and AAm are first dissolved in deionized water at 90 °C to obtain a homogeneous precursor solution, after which APS and MBAA are added to initiate and crosslink the polymer network. The precursor is degassed before casting to minimize trapped bubbles and the resulting internal defects. Under 365 nm UV irradiation, AAm is polymerized to form a covalently crosslinked PAM framework. The macroscopic evolution of the precursor during UV irradiation is shown in Figure S1. The UV−polymerized gel is subsequently frozen at −20 °C for 12 h and thawed at room temperature for 2 h, and the corresponding frozen and thawed states are shown in Figure S2. The freeze−thaw treatment promotes PVA−chain reorganization within the preformed PAM matrix, thereby generating a physically crosslinked PVA network interpenetrated with the PAM covalent scaffold. This appearance change is consistent with the formation of PVA−rich microcrystalline domains and enhanced physical associations within the hydrogel network. This process generated a physically crosslinked PVA network interpenetrating the PAM covalent scaffold (Figure 1b). In the single PAM hydrogel, deformation is mainly accommodated by stretching of covalent chains, and the absence of sufficient reversible energy dissipation units can lead to stress concentration, network fatigue, and unstable electromechanical output during cyclic loading. In the PVA/PAM hydrogel, the PAM network preserves the macroscopic geometry, whereas PVA microcrystalline domains and hydrogen bonds function as dynamic sacrificial junctions. During bending or stretching, these physical associations can dissociate to dissipate mechanical energy and then partially reform after unloading, which improves toughness, stretchability, and sensing repeatability. The hydrated double network also provides deformation−dependent conductive pathways. When the hydrogel sensor is attached to the hand or wrist, joint motion elongates the conductive path and changes the effective cross−sectional area, causing a measurable resistance variation that can be correlated with finger bending, wrist movement, and grip training. These rehabilitation−related signals can be further transmitted to an intelligent assessment platform, enabling rehabilitation−oriented hand−motion monitoring and data−driven motion recognition.

**Figure 2:**
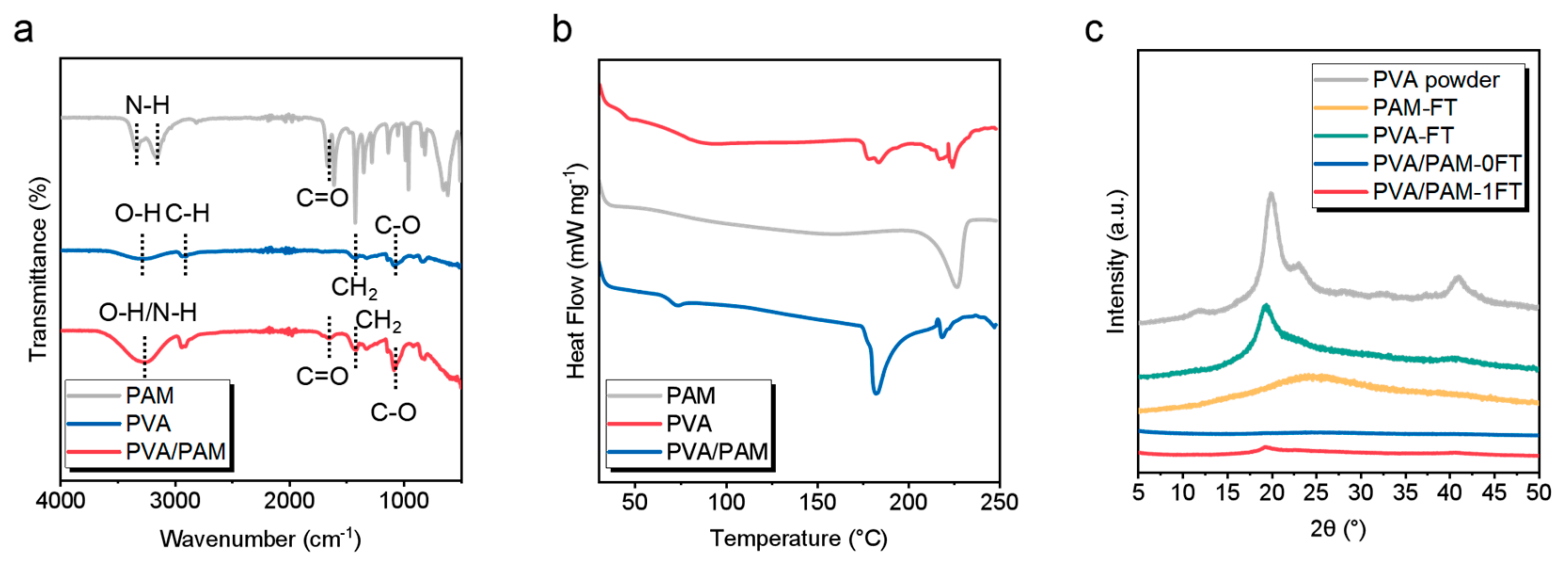
Chemical, thermal, and crystalline evidence of PVA/PAM double−network formation. (**a**) FTIR spectra of PAM, PVA, and PVA/PAM hydrogels. (**b**) DSC curves of PAM, PVA, and PVA/PAM hydrogels. (**c**) XRD patterns of PVA powder, PAM−FT, PVA−FT, PVA/PAM−0FT, and PVA/PAM−1FT.

To elucidate the molecular interactions and freeze−thaw−induced structural evolution of the PVA/PAM network, FTIR and DSC analyses were performed on PAM, PVA, and PVA/PAM hydrogels. XRD measurements were further conducted on PVA powder, freeze−thawed PAM (PAM−FT), freeze−thawed PVA (PVA−FT), PVA/PAM before freeze−thaw treatment (PVA/PAM−0FT), and PVA/PAM after one freeze–thaw cycle (PVA/PAM−1FT). The FTIR spectra first reveal the molecular interactions between the two polymer networks (Figure 2a). PAM exhibits characteristic N–H stretching bands at 3333.8 and 3160.8 cm^−1^ and an amide C=O band at 1667.6 cm^−1^, whereas PVA exhibits an O–H stretching band at 3286.1 cm^−1^. In PVA/PAM, the O–H/N–H band shifts to approximately 3275.5 cm^−1^ and the amide C=O band shifts to approximately 1649.8 cm^−1^. The corresponding red shifts indicate a substantially altered hydrogen−bonding environment between PVA and PAM.

Consistent with the FTIR results, DSC analysis shows pronounced changes in the thermal behavior after formation of the double network (Figure 2b). The high−temperature endothermic transition of PVA shifts to a lower temperature and becomes substantially weaker after incorporation into PVA/PAM, indicating that the thermal environment and chain organization of PVA are strongly modified by the surrounding PAM network.

To directly determine whether freeze−thaw treatment induces PVA chain ordering, XRD analysis was further performed (Figure 2c). PVA powder and PVA−FT exhibit a characteristic PVA−associated diffraction feature at approximately 19–20°, whereas PAM−FT remains predominantly amorphous. PVA/PAM−0FT shows only a weak contribution in this region, while a distinct diffraction feature appears after one freeze−thaw cycle. Quantitative peak deconvolution shows that the apparent crystalline−area fraction increases from 3.68% to 15.87%. These results directly demonstrate that freeze−thaw treatment promotes PVA chain ordering and the formation of PVA−rich microcrystalline domains within the preformed PAM network.

**Figure 3:**
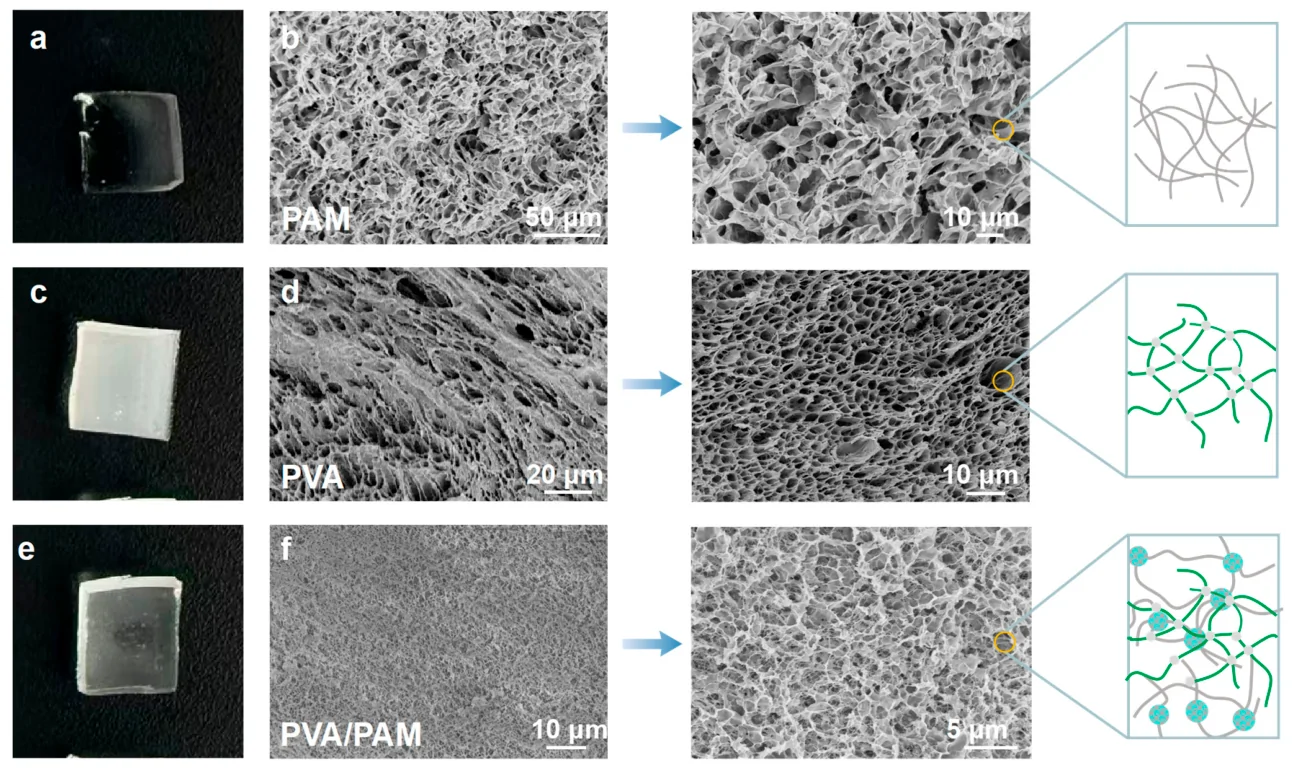
Morphological evolution of the PVA/PAM double−network hydrogel. (**a**) Photograph of PAM hydrogel. (**b**) SEM images and schematic structure of PAM hydrogel. (**c**) Photograph of PVA hydrogel. (**d**) SEM images and schematic structure of PVA hydrogel. (**e**) Photograph of PVA/PAM hydrogel. (**f**) SEM images and schematic structure of PVA/PAM hydrogel.

After confirming the formation of hydrogen bonded PVA and PAM chains at the molecular level, the macroscopic appearance and internal morphology of the hydrogels were examined to determine how this network reconstruction translated into structural organization. The PAM hydrogel appears transparent and soft, indicating a water−rich polymer matrix, while the corresponding scanning electron microscope (SEM) image shows an irregular and relatively open porous skeleton with thin pore walls (Figure 3a,b). At higher magnification, the PAM network remains loose and randomly entangled, which is consistent with a single covalent network that can preserve hydrogel integrity but lacks sufficient secondary junctions to resist local deformation. The schematic beside the SEM image represents this simple chain entanglement, in which polymer strands are connected mainly through sparse covalent crosslinking points. The PVA hydrogel shows a more opaque white appearance, which can be associated with PVA chain aggregation and crystalline domain formation during freezing and thawing. The SEM image reveals an oriented and denser porous morphology compared with PAM, and the enlarged view shows a more continuous pore wall structure with smaller and more regular pores (Figure 3c,d). This morphology reflects the physical network generated by PVA crystallites and hydrogen bonding, as illustrated by the green chain model in the corresponding schematic. The PVA network therefore provides stronger physical association than PAM alone, but its structural contribution remains dominated by one type of physical interaction. The PVA/PAM hydrogel shows a translucent and integrated bulk appearance, suggesting that the two polymer components are incorporated into a continuous hydrogel rather than separated phases (Figure 3e). The SEM images show a markedly more compact and homogeneous porous architecture, with a finer pore size and a highly interconnected pore wall network (Figure 3f). This refined morphology indicates that the preformed PAM covalent network confines PVA crystallization during freezing and thawing, while PVA microcrystalline domains and hydrogen bonds further reinforce the internal skeleton. The schematic on the right visualizes this interpenetrated structure, where grey PAM chains maintain the permanent framework and green PVA chains, crystalline domains, and hydrogen−bonded regions introduce reversible physical junctions. Such a compact porous double network can distribute stress more uniformly, suppress pore collapse under deformation, and provide more stable pathways for deformation−induced resistance changes. The SEM images provide a comparative view of the freeze−dried cross−sectional architectures of the three hydrogels. Compared with the corresponding single−network controls, PVA/PAM exhibits a more compact and interconnected porous morphology, which is consistent with the enhanced intermolecular interactions and PVA microcrystalline−domain formation identified by FTIR, DSC, and XRD analyses. Although freeze−drying may inevitably modify the original hydrated morphology, the SEM observations provide complementary morphological evidence for the structural evolution of the hydrogel network. Therefore, the formation of the PVA/PAM double−network structure is supported by mutually consistent evidence across different characterization scales: FTIR reveals the reconstructed hydrogen−bonding interactions between PVA and PAM, DSC demonstrates the altered chain organization and thermal behavior, XRD directly confirms freeze−thaw−induced PVA microcrystalline domains, and SEM illustrates the corresponding morphological evolution of the freeze−dried network.

**Figure 4:**
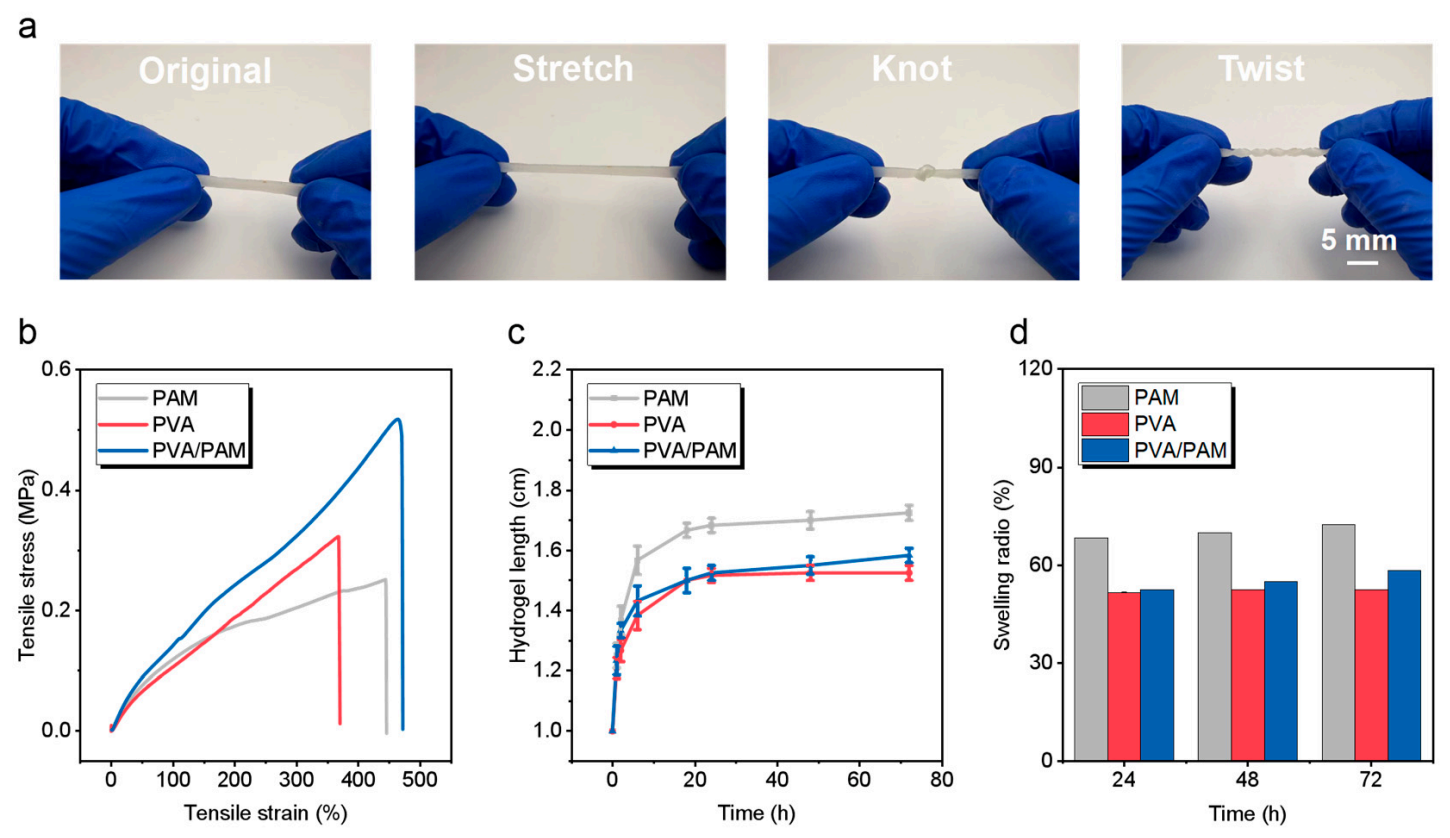
Mechanical properties and swelling stability of the hydrogels. (**a**) Macroscopic deformability of the PVA/PAM hydrogel. (**b**) Tensile stress−strain curves. (**c**) Time−dependent dimensional changes of the hydrogels during swelling. (**d**) Swelling ratio comparison. Data are presented as mean ± SD from n = 7 independently prepared samples.

To correlate the compact double network architecture with macroscopic durability, the deformability, tensile response, and swelling stability of the hydrogels were systematically evaluated. The PVA/PAM hydrogel strip can be stretched, knotted and twisted without any visible fracture or surface damage, showing that the network can accommodate large tensile, bending and torsional deformation (Figure 4a). This is due to the coupled deformation of the PAM covalent scaffold and the PVA physical network. The PAM chains maintain the continuous load transfer during elongation, while the PVA crystallites and hydrogen bonded regions act as reversible junctions that dissipate local stress and delay crack propagation. This structural advantage is further quantified by the tensile stress−strain curves (Figure 4b). A single PAM hydrogel has a relatively low fracture stress, which is attributed to the limited energy dissipation capacity of a sparse covalent network. The PVA hydrogel exhibits a higher stress than PAM but fractures at a lower strain, suggesting that PVA crystallization increases rigidity while restricting chain mobility. By contrast, the PVA/PAM hydrogel reaches the highest tensile stress of approximately 0.52 MPa and maintains an elongation at break of nearly 480%, showing that the two networks do not simply add their individual properties but cooperate during deformation. At the initial stage of stretching, the soft polymer chains extend and align along the loading direction, giving a gradual stress increase. At larger strain, the PVA microcrystalline domains and hydrogen bonds are progressively disrupted, which dissipates mechanical energy and prevents rapid catastrophic failure. This mechanism explains why the PVA/PAM curve rises more steeply at high strain and sustains a larger stress before rupture. The time−dependent dimensional changes indicate that all the samples absorb water quickly in the early stage and then tend to equilibrium, but their final swelling behaviors are quite different (Figure 4c). PAM has the highest dimensional expansion, which is consistent with the loose hydrophilic network and the low resistance to water penetration. PVA presents a lower swelling level due to the presence of crystalline regions and intermolecular hydrogen bonds that restrict the chain relaxation. The PVA/PAM hydrogel presents a moderated swelling behavior, with a lower swelling degree than PAM and a progressive increase with time, which suggests that the interpenetrated network limits the excessive water uptake while keeping the hydration. The swelling ratio statistics at 24, 48 and 72 h further support this interpretation (Figure 4d). PAM presents the highest swelling ratio during the whole test period (more than 70% at 72 h). PVA stays around 50% due to its physically crosslinked crystalline domains. PVA/PAM shows a controlled swelling ratio around 58% after 72 h, slightly higher than PVA but significantly lower than PAM. This result indicates that the PAM network plays a role in water retention and elasticity, whereas the physical network of PVA prevents overexpansion and stabilizes the hydrogel size. This is important for a wearable strain sensor, because over swelling can dilute the conductive pathways, change the initial geometry, and lead to signal drift over repeated uses. In addition to swelling in aqueous environments, dehydration under ambient conditions can also alter the electrical state of an unencapsulated hydrogel sensor. The water loss ratio of the PVA/PAM hydrogel increased progressively with air−exposure time and showed a slower increase at the later stage (Figure S3). In the absence of external deformation, the sensor resistance increased concurrently and gradually approached a relatively stable level (Figure S4). The coupled evolution of water loss and resistance indicates that evaporation reduces the amount of mobile water within the polymer network and restricts ion transport, thereby increasing the electrical resistance. To further clarify the origin of electrical conduction, the baseline resistance and conductivity of PAM, PVA, and PVA/PAM were compared (Table S1). All three hydrogels exhibited measurable conductivity despite the absence of conductive fillers or intentionally added electrolytes, indicating that electrical transport originates primarily from mobile ionic species within the hydrated polymer network. The initial conductivity of PVA/PAM was not higher than that of the corresponding single−network controls, suggesting that the role of the double−network structure is not to increase the number of charge carriers.

The strong dependence of electrical transport on hydration was further demonstrated by controlled dehydration. With progressive water loss, the resistance increased markedly, whereas severe dehydration caused a substantial deterioration in conductivity. Notably, PVA/PAM retained a considerably larger fraction of its conductivity than the single−network controls, indicating that the interpenetrating network more effectively preserves the hydrated ion−transport pathway. This interpretation is further supported by the I–V characteristics (Figure S5), where dehydration results in a clear decrease in conductance. Conversely, short−term washing in DI water decreases the resistance as the network rapidly rehydrates and swells (Figure S6), further demonstrating the close coupling between hydration and ionic transport.

**Figure 5:**
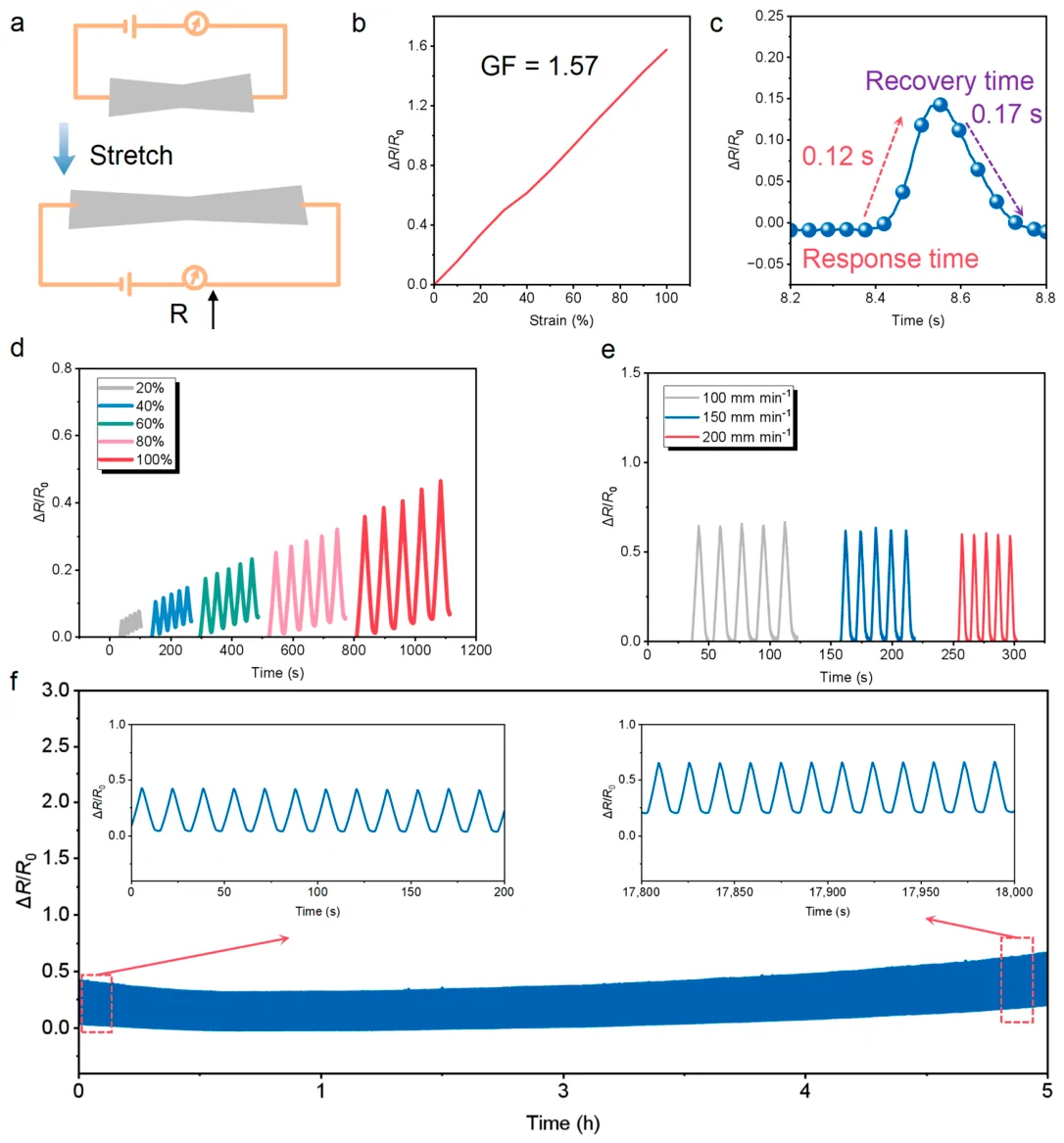
Electromechanical sensing performance of the PVA/PAM hydrogel sensor. (**a**) Resistance variation mechanism under stretching. (**b**) Relative resistance change versus strain. (**c**) Response and recovery behavior. (**d**) Cyclic sensing under different strains. (**e**) Sensing signals at different stretching rates. (**f**) Long−term cyclic stability.

Building on the mechanical compliance and dimensional stability of the PVA/PAM hydrogel, its electromechanical response was evaluated to determine whether the double network could serve as a reliable strain sensing layer under repeated deformation. The resistance signals were acquired using a Keithley 2450 in a two−wire measurement configuration (Figure S7). The sensing principle is described by the resistance change of the hydrogel during stretching (Figure 5a). The measured resistance increases when the hydrogel strip is under tensile strain due to the decrease of the effective cross−sectional area and the longer ion transport pathway. As shown in Figure 5b, the relative change in resistance increases approximately linearly with tensile strain over the investigated range of 10–100%. The gauge factor, calculated from the slope of the ΔR/R_0_−strain relationship, is 1.57, demonstrating a clear and continuous strain−dependent electrical response of the PVA/PAM hydrogel. The increase in resistance arises from the elongation of the ion−transport pathway and the reduction in the effective cross−sectional area during stretching, thereby converting mechanical deformation into a measurable electrical signal. The response and recovery times of the sensor are 0.12 and 0.17 s, respectively, which indicates that the hydrated polymer network can quickly convert the loading and unloading into resistance signals (Figure 5c). Such fast kinetics are important for tracking transient joint movements during rehabilitation. The cyclic stretching at strains from 20% to 100% generates well−separated resistance peaks, and the peak amplitude increases with applied strain (Figure 5d). The repeatable peak shape within each strain level indicates that the PVA microcrystalline domains and hydrogen bonded junctions can withstand repeated dissociation and reconstruction without causing obvious signal distortion. The sensor was further tested under different stretching rates (Figure 5e). The resistance signals remain periodic and distinguishable at 100, 150, and 200 mm min^−1^, indicating that the electromechanical response is not strongly rate dependent within the tested range. This behavior suggests that ion redistribution and polymer chain recovery occur fast enough to follow dynamic deformation. During the long−term durability test, the PVA/PAM hydrogel sensor was subjected to 1000 consecutive loading−unloading cycles over approximately 5 h (Figure 5f). The baseline resistance exhibited a progressive upward shift during prolonged cyclic deformation. Importantly, however, the strain−induced peak−to−baseline amplitude remained well preserved throughout the test, without progressive attenuation of the dynamic response. Analysis of successive cycle windows further revealed only limited cycle−to−cycle variation in peak amplitude, indicating that the repeated strain−induced resistance modulation remained reproducible despite the gradual baseline evolution. Therefore, the long−term response is characterized primarily by baseline drift rather than by progressive loss of strain sensitivity or failure of the conductive pathway. The origin of the baseline drift was further examined by humidity−dependent resistance measurements. As shown in Figure S8, the baseline resistance decreases continuously with increasing relative humidity, confirming the strong dependence of ionic transport on the hydration state of the hydrogel. Therefore, gradual changes in hydration during prolonged operation can contribute substantially to the baseline evolution observed. To further evaluate whether the retained electrical response was accompanied by mechanical stability, cyclic loading–unloading measurements were performed under the same post−drying condition (Figure S9a,b). The representative stress–strain loops remained highly comparable at different cycle numbers, while the elastic recovery rate remained high and the energy−loss coefficient showed no progressive deterioration. These results demonstrate that the PVA/PAM hydrogel retains both mechanical recovery and reproducible electromechanical response during prolonged cyclic operation.

**Figure 6:**
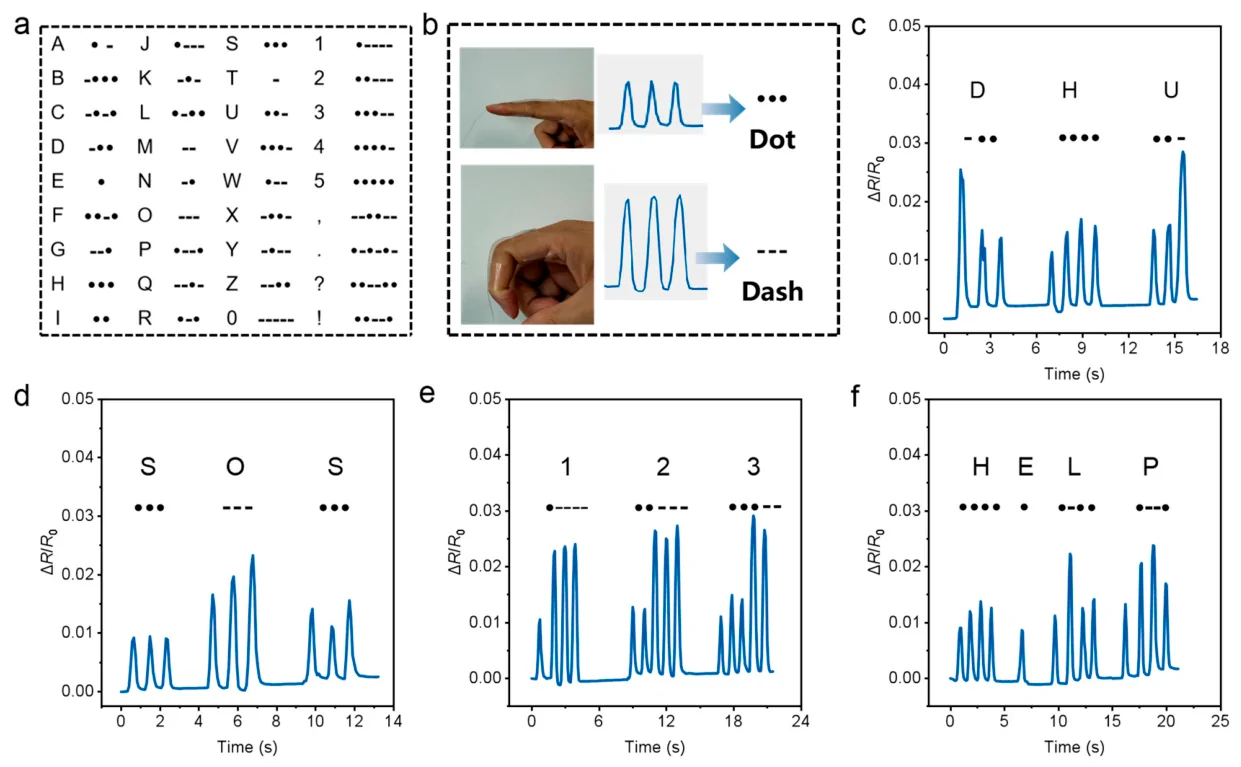
Morse−code information transmission based on finger motion. (**a**) Morse−code library. (**b**) Definition of dot and dash signals. (**c**) Encoding of DHU. (**d**) Encoding of SOS. (**e**) Encoding of 123. (**f**) Encoding of HELP.

Having established the rapid and repeatable electromechanical conversion of the PVA/PAM hydrogel sensor, the device was further used as a motion−encoded interface for rehabilitation−related information transmission. The standard morse code library was introduced to establish the relationship between alphabetic or numeric information and electrical pulse sequences (Figure 6a). This coding rule provides a simple framework in which short and long signals can be translated into readable messages. Related self−powered core−spun yarn sensors have likewise converted finger−triggered electrical outputs into morse−code messages, supporting the feasibility of wearable human−input communication [51]. The hydrogel sensor was attached to the index finger to define the basic input units (Figure 6b). A rapid finger movement generated a narrow resistance pulse and was assigned as a dot, whereas a prolonged bending movement produced a broader signal and was assigned as a dash. As both units are driven by voluntary finger movement, this encoding process can be directly used in hand rehabilitation training, where repetitive extension and flexion are widely used to evaluate motor control, joint mobility, and patient participation. The resistance peaks rapidly recovered to the baseline after each movement, which indicated that the double network hydrogel exhibited stable electrical recovery during repeated finger actuation. The encoded signal corresponding to “DHU” was reconstructed from a sequence of dots and dashes (Figure 6c). The separated pulse clusters show clear temporal boundaries between different letters, allowing each character to be distinguished without overlap. The sensor encoded the emergency message “SOS” (Figure 6d). The three short pulses, three long pulses, and three short pulses were clearly resolved in the resistance trace, confirming that the sensor can convert a simple rehabilitation movement into a recognizable distress signal. The numerical sequence “1 2 3” was further generated, showing that the sensor can transmit not only letters but also quantitative information (Figure 6e). This capability is relevant for rehabilitation scenarios in which patients may need to report training counts, pain levels, or exercise stages through limited finger motion. The more complex word “HELP” was encoded with multiple letters and unequal pulse intervals (Figure 6f). Throughout the whole sequence, the signal showed discrete peak patterns, indicating that the sensor can sustain the readability of signals over longer information transfer. The capability to transform controlled finger movements into digital messages links strain sensing with interactive rehabilitation, enabling patients to communicate basic needs and training states through simple hand motions while the electrical signals remain suitable for remote monitoring and intelligent assessment. Therefore, the morse−code demonstration is regarded as an auxiliary communication function rather than a rehabilitation−assessment metric, providing a proof−of−concept approach for transmitting assistance or emergency messages through simple voluntary finger movements.

**Figure 7:**
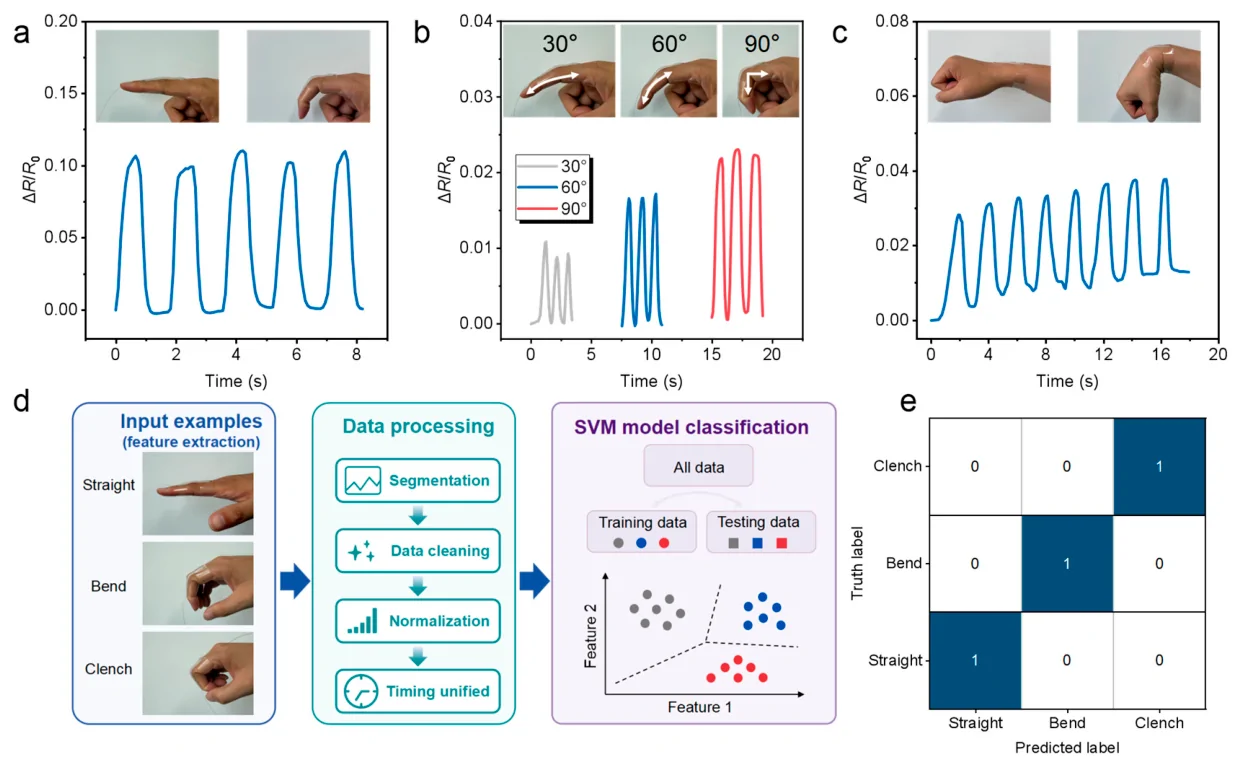
Rehabilitation−oriented hand motion monitoring and machine−learning−assisted recognition. (**a**) Finger−bending sensing. (**b**) Finger−bending angle discrimination. (**c**) Wrist motion sensing. (**d**) Machine−learning recognition workflow. (**e**) SVM classification result.

To move from motion−encoded communication toward rehabilitation assessment, the PVA/PAM hydrogel sensor was attached to representative hand and wrist joints and coupled with a supervised learning workflow for action recognition. The sensor was fixed on the index finger to record repeated transition between the straight state and the bent state (Figure 7a). Each bending event produced a sharp increase in ΔR/R_0_, followed by a rapid return to the baseline after finger extension, indicating that the hydrogel could conform to local skin deformation and reversibly transduce joint flexion into electrical output. The nearly identical peak height and waveform in the multiple cycles showed the capability of the sensor to sustain stable interfacial contact and repeatable resistance modulation during the dynamic finger rehabilitation movement. The same sensing principle was used to distinguish different finger−bending angles (Figure 7b). The resistance amplitude increased from 30° to 60° and then to 90°, which is consistent with the larger tensile deformation imposed on the hydrogel at higher joint curvature. The three signal groups are temporally separated and show clear differences in peak intensity, demonstrating that the sensor is not limited to binary detection of bending and extension but can resolve graded joint motion. This capability is important for rehabilitation training because the recovery of hand function is often evaluated by the controllable range of motion rather than by a single movement state. The sensor was further applied to repeated wrist flexion (Figure 7c), where periodic resistance variations remained clearly distinguishable during continuous motion. This result demonstrates that the sensor can follow larger joint deformation beyond finger flexion and supports its use for rehabilitation−oriented motion monitoring. Because wearable sensors may be exposed to sweat or other aqueous ionic environments during use, the sensing response was further compared in air and physiological saline (Figure S10a,b). Immersion in physiological saline produced an evident shift in the resistance baseline, confirming that the absolute resistance of the ionic hydrogel is sensitive to the surrounding hydration and ionic environment. Importantly, however, the peak−to−baseline resistance modulation induced by repeated deformation remained comparable in air and physiological saline, and clearly distinguishable periodic responses were retained. Thus, the ionic environment primarily affects the baseline electrical state rather than substantially attenuating the deformation−induced resistance response. Because the sensor is intended for direct skin−contact wearable applications, its cytocompatibility was further evaluated using an extract−based CCK−8 assay with NIH/3T3 fibroblasts (Figure S11). The metabolic activity of cells exposed to the PVA/PAM hydrogel extract was comparable to that of the untreated control, with no statistically significant difference between the two groups. This result indicates that no detectable acute cytotoxicity was induced by leachable components from the PVA/PAM hydrogel under the tested extraction conditions, supporting its cytocompatibility for skin−contact sensing applications. Comparable fibrous thermoelectric fabrics have also enabled physiological and joint-motion monitoring together with wireless sign-language interaction, demonstrating the broader utility of compliant wearable sensing architectures [52]. Furthermore, the electrical signals from three hand states were converted into a machine learning input pipeline (Figure 7d). Straight, bend and clench were chosen as representative rehabilitation gestures to reflect different levels of tendon movement, joint flexion and grip actuation. The quantitative grip−related data are shown in Figure S12. The workflow starts with feature extraction from the raw resistance signals, followed by segmentation, data cleaning, normalization and timing unification. Segmentation is to extract each movement cycle from the continuous time series. Data cleaning is to remove abnormal fluctuations caused by motion artifacts or temporary contact instability. Normalization is to mitigate the amplitude differences among trials and enhance the comparability among samples. Timing unification aligns signal length and temporal structure so that the extracted features can be consistently processed by the classifier. After preprocessing, all data were divided into training and testing subsets and introduced into an SVM model. The SVM scheme maps the extracted motion features into a multidimensional feature space and identifies decision boundaries that separate different gesture classes. This design converts the hydrogel from a passive strain sensor into a data−driven recognition interface, allowing similar but mechanically distinct rehabilitation movements to be classified from their resistance signatures. Figure 7e shows that the signal patterns generated by the three hand states are sufficiently distinct after preprocessing and feature extraction. The SVM classifier achieved an overall accuracy of 100%. To further clarify the performance positioning of the present sensor, a quantitative comparison with representative recent hydrogel strain sensors and machine−learning−assisted wearable sensors is provided in Table S2 [53,54,55,56,57,58,59,60,61]. The present PVA/PAM system provides a balanced combination of mechanical robustness, rapid electromechanical response, cyclic durability, and motion−recognition capability, although it does not exhibit the highest value in every individual metric. These comparisons highlight the integrated sensing performance of the present platform rather than superiority in a single parameter.

## 3. Conclusions

In this work, a PVA/PAM double−network hydrogel strain sensor was developed through UV−induced PAM covalent crosslinking and freeze–thaw−induced PVA physical crosslinking for wearable rehabilitation monitoring. The PAM network provided a continuous load−bearing framework, while PVA microcrystalline domains and hydrogen bonds acted as reversible physical junctions for energy dissipation and structural recovery. The combined FTIR, DSC, and XRD results support the formation of the PVA/PAM double−network structure, while SEM provides complementary information on the freeze−dried morphology. Benefiting from this structure, the hydrogel exhibited good deformability, a tensile strength of approximately 0.52 MPa, an elongation at break of approximately 480%, and a controlled swelling ratio of approximately 58% after 72 h. As a strain sensor, it showed gauge factors of 1.57, a response time of 0.12 s, and a recovery time of 0.17 s, together with stable resistance signals under cyclic strain and different deformation rates. The sensor further enabled finger bending, wrist motion, grip training, morse−code information transmission, and SVM−based recognition of straight, bend, and clench states. These results demonstrate the potential of the PVA/PAM hydrogel as a wearable platform for rehabilitation−oriented hand−motion monitoring and proof−of−concept motion recognition.

## 4. Materials and Methods

### 4.1. Materials

Polyvinyl alcohol (PVA−1799) was purchased from Aladdin Biochemical Technology Co., Ltd., Shanghai, China. Acrylamide (AAm), ammonium persulfate (APS), and N, N’−methylenebisacrylamide (MBAA) were obtained from Shanghai Titan Scientific Co., Ltd., Shanghai, China. Deionized (DI) water with a resistivity of 18.2 MΩ·cm prepared in the laboratory was used throughout the experiments. All chemical reagents were of analytical grade and used as received without further purification.

### 4.2. Preparation of Hydrogel

PVA hydrogel: PVA−1799 was dissolved in deionized water under magnetic stirring at 90 °C to prepare a 10 wt% PVA solution. After complete dissolution, the solution was cooled down, degassed under vacuum, and poured into molds for freezing.

PAM hydrogel: 1.5 g of AM was dissolved in 10 mL deionized water under stirring. 36 mg APS and 3 mg MBAA were then added and mixed homogeneously. The mixture was irradiated under UV light for 1 h to accomplish polymerization.

The PVA/PAM double−network (DN) hydrogels were prepared via a strategy combining in situ ultraviolet (UV) light−initiated polymerization and physical freeze–thaw cycles. Briefly, 2 g of PVA monomers was dispersed in 20 mL of DI water and magnetically stirred at 90 °C until complete dissolution to yield a homogeneous and transparent precursor solution. After the solution cooled to room temperature, 72 mg of the free−radical initiator APS, 10 mg of the chemical crosslinker MBAA and 3 g of AAM were sequentially added. The mixture was continuously stirred under dark conditions until thoroughly mixed, followed by ultrasonic degassing to remove tiny air bubbles. The degassed precursor solution was then cast into a mold with dimensions of 5 cm × 5 cm × 2 mm and irradiated under 365 nm UV light for 1 h to induce the free−radical polymerization of AAm, forming the covalently crosslinked first PAM network. Following the UV polymerization, the hydrogel was frozen at −20 °C for 12 h to promote the crystallization of PVA chains during the phase separation process. Finally, the sample was transferred to room temperature (37 °C) and allowed to thaw slowly for 2 h. In this process, the microcrystalline domains and intermolecular hydrogen bonds of PVA established the physically crosslinked second network, ultimately yielding a dense and robust PVA/PAM double−network hydrogel.

### 4.3. Characterization

#### 4.3.1. Structural Characterization

Fourier transform infrared (FTIR) spectra were recorded using an FTIR spectrometer (Antaris II, Thermo Fisher Scientific, Waltham, MA, USA) in the wavenumber range of 4000–500 cm^−1^ to investigate the functional groups within the hydrogel network and the reconstructed hydrogen−bonding interactions between the double networks.

To examine the internal morphology, scanning electron microscopy (SEM) was employed to characterize the microscopic pore structure of the hydrogels. The hydrogel samples were swollen in DI water for 24 h and subsequently freeze−dried. After 48 h of freeze−drying, the dried samples were fractured in liquid nitrogen. The cross−sections were sputter−coated with a thin layer of gold using an ion sputterer (ISC 150, Shenzhen SuPro Instruments Co., Ltd., Shenzhen, China) to enhance electrical conductivity before observation.

#### 4.3.2. Mechanical Testing

The mechanical properties of the hydrogels were quantitatively evaluated using a universal tensile testing machine (YG(B) 026G−500, DARONG, Wenzhou, China). Before testing, the as−prepared hydrogels were cut into rectangular strips with an effective dimension of 4 cm in length and 1 cm in width. Tensile tests were performed at a speed of 20 mm min^−1^ until sample fracture. The corresponding stress–strain curves were plotted and analyzed using Origin software.

#### 4.3.3. Differential Scanning Calorimetry Testing

To analyze the thermodynamic behavior differences and molecular chain segment interactions between the single−component polymers and the double−network composite system, differential scanning calorimetry (DSC) analysis was performed using a DSC instrument (DSC 8500, PerkinElmer, Inc., Waltham, MA, USA). Five mg of the thoroughly dried hydrogel sample was accurately weighed and sealed in an aluminum pan. Under a continuous nitrogen atmosphere, the sample was heated to 250 °C at a constant heating rate of 10 °C min^−1^. The heat flow curve was continuously recorded to selectively analyze the characteristic melting peak of PVA and the newly formed endothermic peak in the PAM−PVA system, resulting from strong hydrogen−bonding reconstruction.

#### 4.3.4. Swelling Testing

To evaluate the swelling performance of the material in specific environments, a dimensional measurement method was used to monitor the swelling kinetics of the hydrogels. First, the initial length of the hydrogel sample was measured using a vernier caliper and recorded as L_0_. Subsequently, the sample was completely immersed in DI water. At designated time intervals, the sample was removed, gently wiped with absorbent filter paper to remove surface free water, and its transient dimension was immediately measured and recorded as L_t_. This procedure was repeated until the dimension of the hydrogel showed no significant changes. The swelling ratio (%) of the samples was calculated according to the following equation:

$$\mathrm{Swelling}\;\mathrm{ratio}=\frac{L_{t}-L_{0}}{L_{0}}\times 100\%$$

#### 4.3.5. Electrical Characterization

The baseline resistance of PAM, PVA, and PVA/PAM hydrogels was measured using a Keithley 2450 source meter in a two−wire configuration. The depth of the electrode insertion is approximately 0.5 cm, and the distance between the two electrodes is approximately 3 cm. The conductivity was calculated from the measured resistance, electrode spacing, and hydrogel cross−sectional dimensions. For dehydration measurements, the sample mass, dimensions, and resistance were recorded before and after controlled heating. I–V characteristics were measured using the same electrode configuration. For short−term washing measurements, the hydrogels were immersed in DI water for predetermined periods, gently blotted to remove surface water, and immediately subjected to resistance measurements.

#### 4.3.6. Extract−Based Cytocompatibility Assay

The PVA/PAM hydrogel was extracted in complete cell−culture medium at a relatively high material−to−medium ratio of 400 mg mL^−1^ at 37 °C for 24 h. The resulting extract was sterilized through a 0.22 μm membrane and subsequently used for cell exposure. NIH/3T3 cells were allowed to adhere for 6 h and were then cultured with the hydrogel extract for a further 18 h, followed by CCK−8 analysis.

## Data Availability

All data, models, and codes generated or used during the study are Available from the corresponding author by request.

## Supplementary Materials

The following supporting information can be downloaded at: https://www.mdpi.com/article/10.3390/gels12080730/s1, Figure S1: UV−induced gelation of the PVA/PAM system at different times; Figure S2: Freeze−thaw treatment; Figure S3: Water−loss ratio as a function of air−exposure time; Figure S4: Resistance variation of the hydrogel sensor during air exposure without applied deformation; Figure S5: I−V characteristics of the PVA/PAM hydrogel before and after dehydration; Figure S6: Resistance variation of PAM, PVA, and PVA/PAM hydrogels during washing; Figure S7: Schematic of the resistance measurement setup; Figure S8: Resistance response of the PVA/PAM hydrogel at different relative humidity levels; Figure S9: Cyclic mechanical recovery and energy-dissipation behavior of the PVA/PAM hydrogel; Figure S10: Cyclic sensing responses of the PVA/PAM hydrogel in air and physiological saline; Figure S11: Cytocompatibility of the PVA/PAM hydrogel extract toward NIH/3T3 fibroblasts; Figure S12: Quantitative grip-related measurements; Table S1: Electrical properties of PAM, PVA, and PVA/PAM hydrogels during dehydration; Table S2: Comparison of the mechanical, sensing, environmental, and motion-recognition performance of representative hydrogel strain sensors. Refs. [53,54,55,56,57,58,59,60,61] are cited in the Supplementary Materials.
