# Supplementary material for: Dual−Network PVA/PAM Hydrogel Strain Sensor for Machine−Learning−Assisted Rehabilitation−Oriented Hand Motion Monitoring
Source: Gels. 2026 Aug 17;12(8):730. doi: 10.3390/gels12080730 (PMC13512588; doi:10.3390/gels12080730)
Supplement: Supplementary file 1 [file gels-12-00730-s001.zip › gels-4469752-supplementary.pdf]

Supplementary Information

Dual–Network PVA/PAM Hydrogel Strain Sensor for Machine–Learning–Assisted Rehabilitation–Oriented Hand Motion Monitoring

Wendi Liu [1,2,†], Jintao Wang [1,†], Yuanduo Wang [3,†], Zhangqi Xia [1], Ruixin Liu [1], Yixuan Li [2], Xinyang He [1,*] and Hailou Wang [1,*]

[1] National & Local Joint Engineering Research Center of Technical Fiber Composites for Safety and Health, School of Textile & Clothing, Nantong University, Nantong 226019, China
[2] Shanghai Frontiers Science Center of Advanced Textiles, College of Textiles, Donghua University, Shanghai 201620, China
[3] Key Laboratory of Textile Science & Technology, Ministry of Education, College of Textiles, Donghua University, Shanghai 201620, China
* Correspondence: hexinyang@ntu.edu.cn (X.H.); wanghl1130@ntu.edu.cn (H.W.)
† These authors contributed equally to this work.

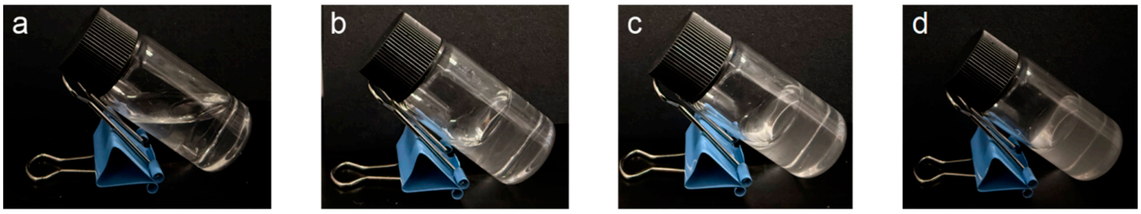

Figure S1. UV-induced gelation of the PVA/PAM system at different times. a 0 min. b 5 min. c 10 min. d 60 min.

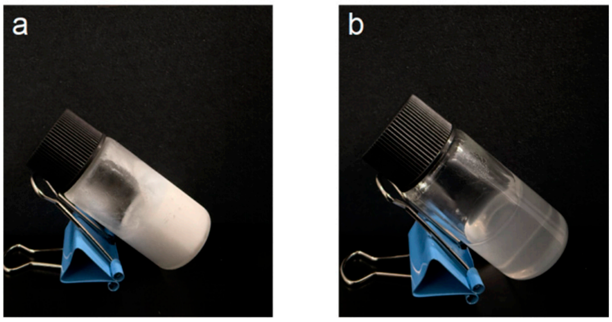

Figure S2. Freeze–thaw treatment. a Frozen state after 12 h at −20 °C. b Thawed state after 2 h at room temperature.

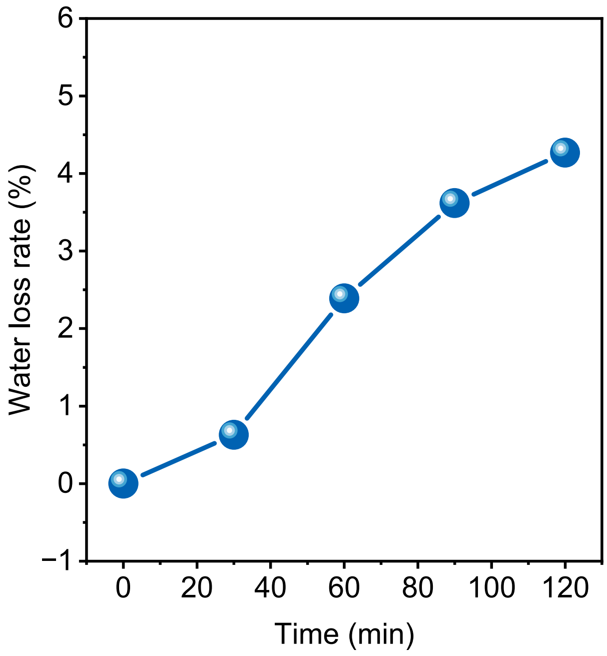

Figure S3. Water-loss ratio as a function of air-exposure time.

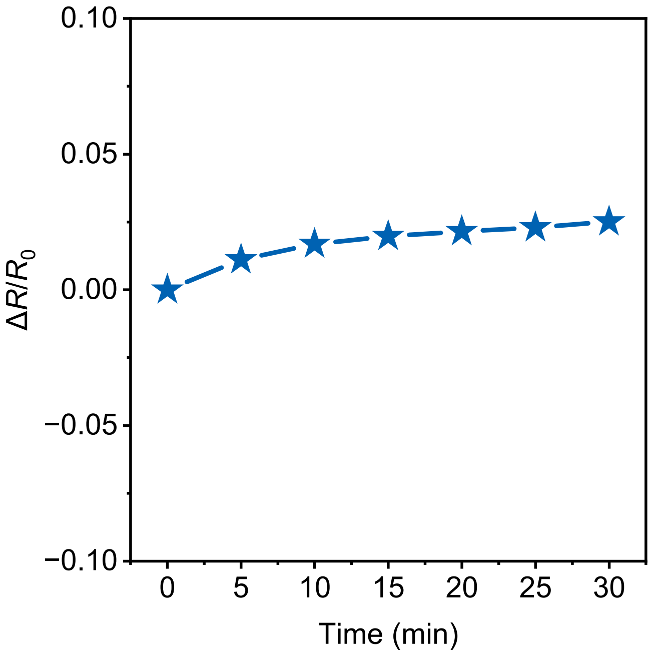

Figure S4. Resistance variation of the hydrogel sensor during air exposure without applied deformation.

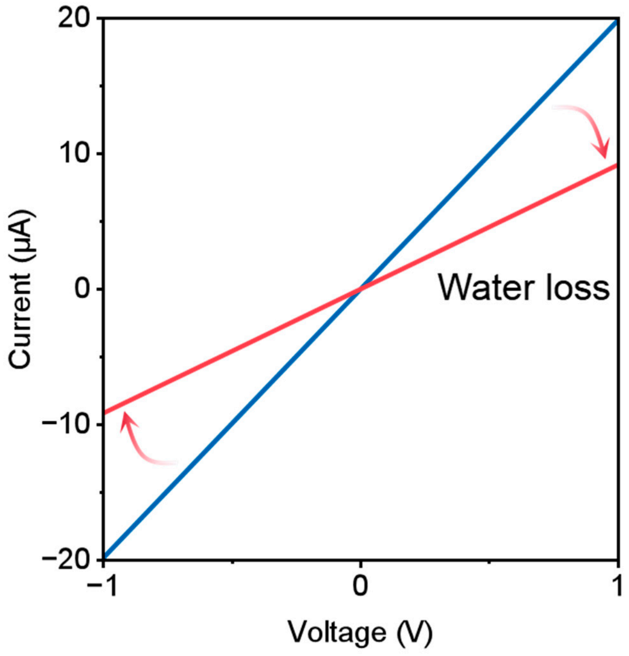

Figure S5. I–V characteristics of the PVA/PAM hydrogel before and after dehydration.

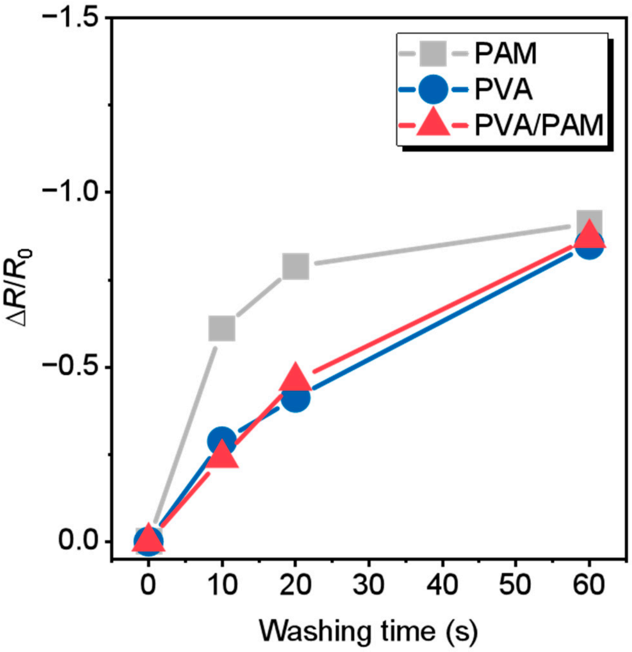

Figure S6. Resistance variation of PAM, PVA, and PVA/PAM hydrogels during washing.

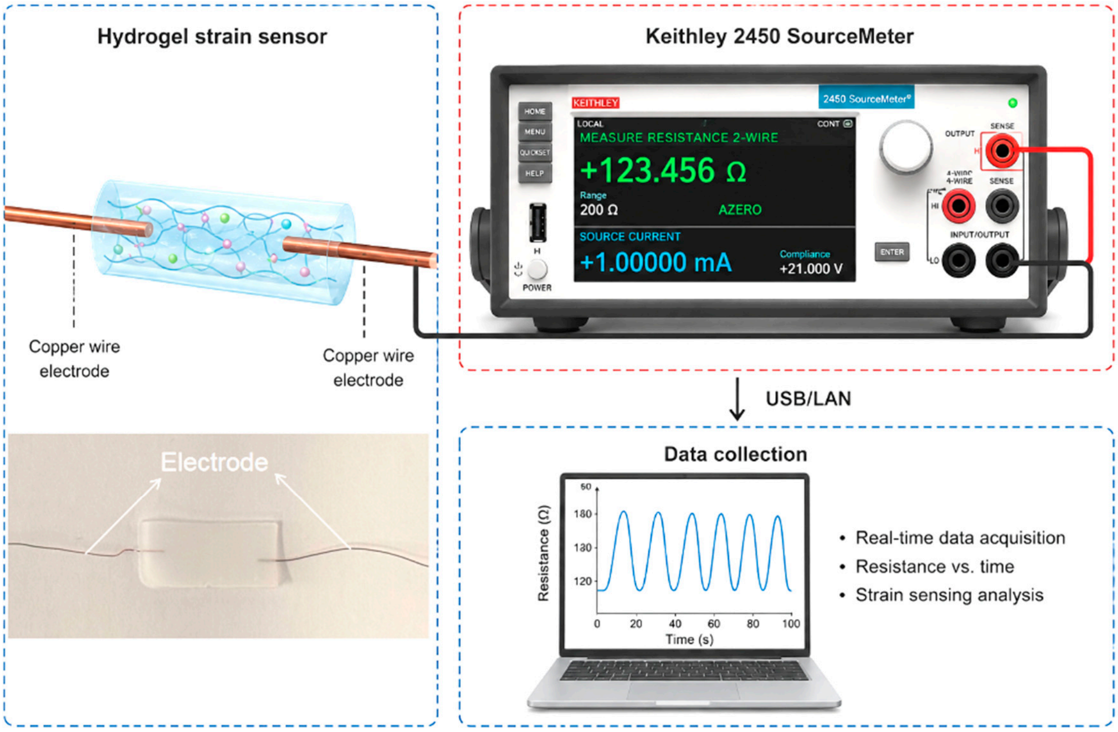

Figure S7. Schematic of the resistance measurement setup.

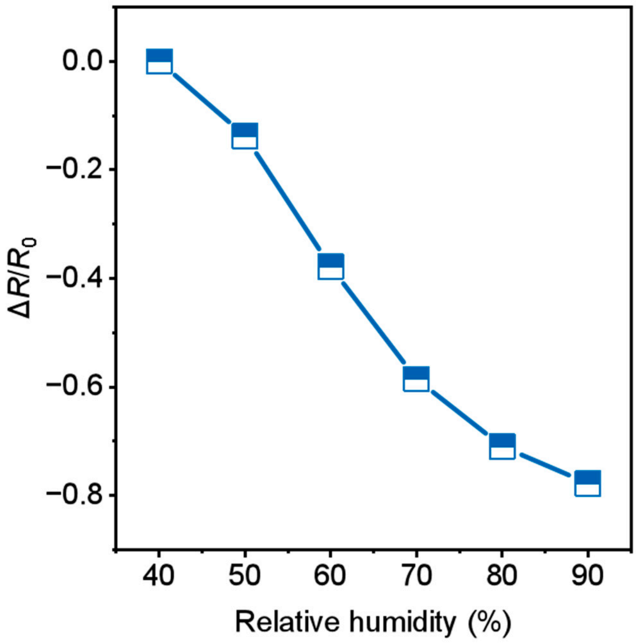

Figure S8. Resistance response of the PVA/PAM hydrogel at different relative humidity levels.

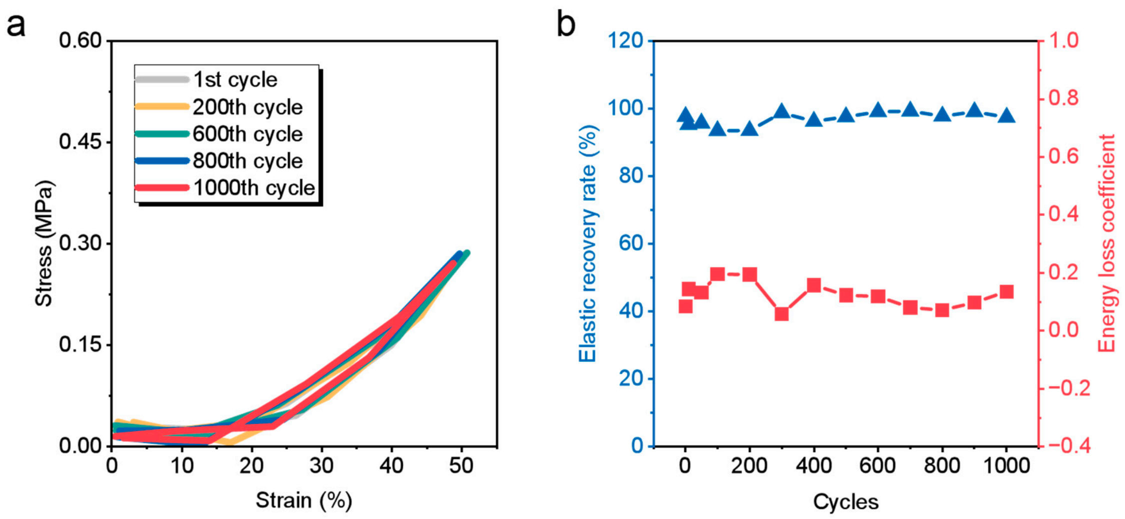

Figure S9. Cyclic mechanical recovery and energy-dissipation behavior of the PVA/PAM hydrogel. a Loading–unloading stress–strain curves at 50% strain. b Elastic recovery rate and energy-loss coefficient as a function of cycle number.

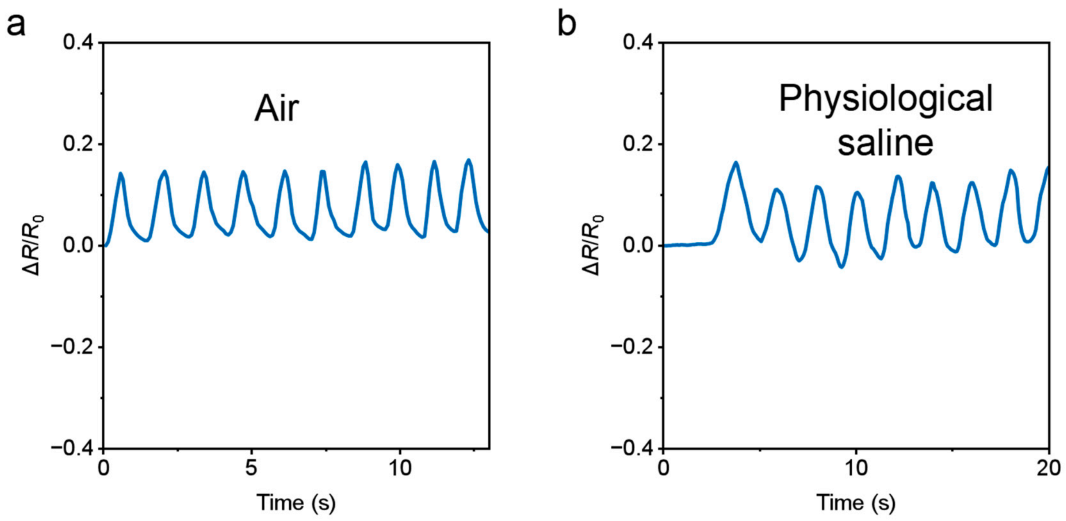

Figure S10. Cyclic sensing responses of the PVA/PAM hydrogel in air and physiological saline. a Air. b Physiological saline.

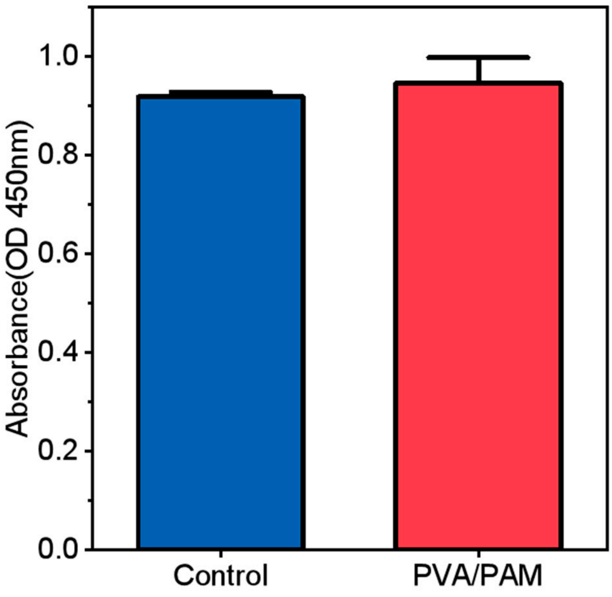

Figure S11. Cytocompatibility of the PVA/PAM hydrogel extract toward NIH/3T3 fibroblasts.

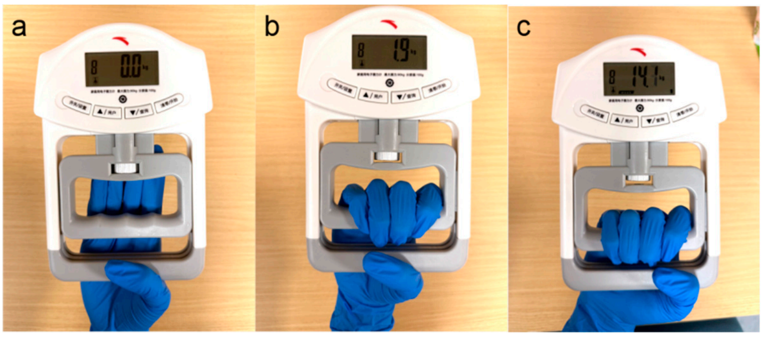

Figure S12. Quantitative grip-related measurements. a Straight. b Bend. c Clench.

Table S1. Electrical properties of PAM, PVA, and PVA/PAM hydrogels during dehydration.

**Sample**	**Condition**	**Mass (g)**	**Mass loss (%)**	R/R_0	**Conductivity (μS cm^{-1})**
PAM	Initial	0.772	0	1.000	11.27
	80 °C, 10 min	0.392	49.22	1.209	11.65
	80 °C, 1 h	0.034	95.60	Overflow	——
PVA	Initial	0.482	0	1.000	9.36
	80 °C, 10 min	0.242	49.79	1.283	10.94
	80 °C, 1 h	0.073	84.85	17.476	0.961
PVA/PAM	Initial	0.706	0	1.000	7.67
	80 °C, 10 min	0.300	57.51	1.257	9.42
	80 °C, 1 h	0.085	87.96	3.010	4.89

Table S2. Comparison of the mechanical, sensing, environmental, and motion-recognition performance of representative hydrogel strain sensors.

Hydrogel	Strength (MPa)	Elongation (%)	GF	Response time (ms)	Cycling durability	Environmental stability	Number of classified gestures	Accuracy (%)	Ref.
PVA/PAM DN	0.2682	2253	2.32	368	30 bending cycles	NR	No ML classification	NR	[1]
BPGPH	0.318	400	0.29	NR	NR	12 days	NR	NR	[2]
MPRS	NR	315	NR	330	250 cycles	NR	No ML classification	NR	[3]
PLM	0.0327	317	NR	NR	1000 cycles	NR	4	100	[4]
LH	0.18	515	2.6	NR	NR	NR	NR	98.08	[5]
PSB–HEC	0.054	NR	1.63	2110	NR	NR	No ML classification	NR	[6]
PAHS	0.490	1140	1.60	155	NR	NR	NR	NR	[7]
PCMF	0.343	1654	1.49	290	500 cycles	NR	10	99.5	[8]
PS–Al^{3+}	9.71	993.53	0.57	403	NR	NR	No ML classification	NR	[9]
PVA/PAM DN	0.52	480	1.57	120	1000 cycles	5 h	3	100	This Work

Abbreviations: DN, double network; BPGPH, BMIM-BF_4/PEDOT:PSS/gelatin/PVA hydrogel; MPRS, MXene/PEDOT:PSS/rGO/silk-fibroin/PVA hydrogel; PLM, PAM/LiCl/MXene hydrogel; LH, low-hysteresis glycerol/LiCl hydrogel; PSB–HEC, poly(sulfobetaine)/hydroxyethyl cellulose hydrogel; PAHS, PVA/PAMAAC/HACC/sodium-citrate hydrogel; PS–Al^{3+}, PAM/SA–Al^{3+} hydrogel.

References

1. Wu, Z. J.; Liu, X. R.; Xu, Q.; Zhang, L. Y.; Abdou, S. N.; Ibrahim, M. M.; Zhang, J.; El-Bahy, Z. M.; Guo, N.; Gao, J. G.; Weng, L.; Guo, Z. H. Poly(Vinyl Alcohol)/Polyacrylamide Double-Network Ionic Conductive Hydrogel Strain Sensor with High Sensitivity and High Elongation at Break. J. Polym. Sci. 2024, 62 (20), 4599-4611.
2. Yang, X.; Zhang, H.; Wang, Z.; Sun, X. P.; Ren, H. C. Skin-Mountable and Self-Healable Hydrogel for Strain Sensing. ACS Appl. Electron. Mater. 2025, 7 (9), 4371-4381.
3. Zheng, Z. J.; Xu, W. J.; Wang, Y.; Xiong, W. J.; Xiong, C. H.; You, L. J.; Wang, S. Y. High-Conductivity and Long-Term Stability Strain Sensor Based on Silk Fibroin and Polyvinyl Alcohol Hydrogels. Mater. Today Commun. 2024, 38, 108465.
4. Hasan, S.; D'Auria, B. G.; Mahmud, M. A. P.; Adams, S. D.; Long, J. M.; Kong, L. X.; Kouzani, A. Z. AI-Aided Gait Analysis with a Wearable Device Featuring a Hydrogel Sensor. Sensors 2024, 24 (22), 7370.
5. Jiang, H. J.; Lai, J. X.; Zhang, P. Q.; Lai, J.; Tu, A. M.; Lai, K.; Xiao, L. Y. Low-Hysteresis Hydrogels with Antidehydration as a Stretchable Strain Sensor for Gesture Recognition. ACS Appl. Polym. Mater. 2024, 6 (19), 11922-11931.
6. Madani, M.; Borandeh, S.; Baniasadi, H.; Basarir, F.; Vapaavuori, J.; Seppala, J.; Niskanen, J. Zwitterionic Cellulose Hydrogels for Flexible Strain Sensors with Enhanced Sensing and Mechanical Performance. ACS Appl. Polym. Mater. 2025, 7 (21), 15100-15110.
7. Liu, P.; Li, F. H.; Gu, Y. X.; Li, X. Y.; Yu, Y. W.; Chen, C. X.; Zhang, H. Z.; Li, Y. H.; Mao, J. P.; Gong, H. Y.; Shan, X. T. Stretchable, Self-Adhesive, and Sensitive Polyacrylamide-Polyvinyl Alcohol Double Network Hydrogels for Flexible Wearable Sensors. Mater. Today Commun. 2024, 41, 110804.
8. Zhou, X.; Liu, Y. S.; Liu, L. T.; Wang, M. C.; Shi, Y. L.; Yu, H. J.; Miao, Y. Q.; Yue, B. B.; Zhu, L. L. Metal-Coordinated Sacrificial-Bond Dual-Network Toughness Architecture: An Ultra-Stretchable and Highly Sensitive Wearable Hydrogel Keyboard for Gesture Recognition. Chem. Eng. J. 2026, 529, 172693.
9. Song, X. C.; Guo, J.; Chen, W.; Liu, M. Y.; Zhang, Y. H.; Xiao, W. H.; Guan, F. C. A High-Strength, Anti-Swelling Sodium Alginate/Polyacrylamide Hydrogel Strain Sensor for Underwater Motion Monitoring and Information Transmission. Gels 2026, 12 (6), 468.
